# Loss of NPM2 expression is a potential immunohistochemical marker for malignant peritoneal mesothelioma: a single-center study of 92 cases

**DOI:** 10.1186/s12957-022-02811-y

**Published:** 2022-10-24

**Authors:** He-liang Wu, Zhi-ran Yang, Yan-dong Su, Ru Ma, Xue-mei Du, Ying Gao, Yan Li

**Affiliations:** 1 Department of Peritoneal Cancer Surgery, Beijing Shijitan Hospital, Peking University Ninth School of Clinical Medicine, Beijing, 100038 China; 2 Department of Peritoneal Cancer Surgery, Beijing Shijitan Hospital, Capital Medical University, Beijing, 100038 China; 3 Department of Pathology, Beijing Shijitan Hospital, Capital Medical University, Beijing, 100038 China

**Keywords:** Malignant peritoneal mesothelioma, Nucleoplasmin 2, QuPath software, Immunohistochemistry, Prognosis

## Abstract

**Background:**

Malignant peritoneal mesothelioma (MPM) is a rare malignant tumor with a high mortality rate and extremely poor prognosis. In-depth pathological analysis is essential to assess tumor biological behaviors and explore potential therapeutic targets of MPM. Nucleoplasmin 2 (NPM2) is a molecular chaperone that binds histones and may play a key role in the development and progression of tumors. This study aimed to analyze the correlation between the expression level of NPM2 and the main clinicopathological characteristics and prognosis of MPM.

**Methods:**

Ninety-two postoperative specimens from MPM patients following cytoreductive surgery were collected. Postoperative specimens were stained with immunohistochemistry. The expression level of NPM2 was quantitatively analyzed by QuPath-0.3.2 software. Univariate and multivariate analyses were conducted to investigate the correlation between NPM2 expression and other conventional clinicopathological characteristics.

**Results:**

Among the 92 MPM patients, there were 47 males (48.9%) and 45 females (51.1%), with a median age of 56 (range: 24–73). There were 70 (76.0%) cases with loss of NPM2 protein expression, 11 (12.0%) cases with low expression, and 11 (12.0%) cases with high expression. Univariate analysis showed that NPM2 protein expression level (negative vs. low expression vs. high expression) was negatively correlated with the following three clinicopathological factors: completeness of cytoreduction (CC) score, vascular tumor emboli, and serious adverse events (SAEs) (all *P* < 0.05). Multivariate analysis showed that NPM2 protein expression level (negative vs. low expression vs. high expression) was independently negatively correlated with the following two clinicopathological factors: CC score [odds ratio (OR) = 0.317, 95% *CI*: 0.317–0.959, *P* = 0.042] and vascular tumor emboli (*OR* = 0.092, 95% *CI* = 0.011–0.770, *P* = 0.028). Survival analysis showed that loss of NPM2 protein expression (negative vs. positive) was associated with poor prognosis of MPM.

**Conclusions:**

Loss of NPM2 expression is a potential immunohistochemical marker for MPM.

## Introduction

Malignant peritoneal mesothelioma (MPM) is a rare malignant tumor arising from peritoneal mesothelium, accounting for 7–30% of all malignant mesothelioma [1]. The World Health Organization (WHO) classifies the histological types of MPM into three major types: epithelioid, sarcomatoid, and biphasic, with epithelioid being most common [2].

Nucleoplasmin (NPM), containing three subtypes NPM1, NPM2, and NPM3, is a class of abundant and widely expressed phosphorylated proteins that can dynamically shuttle across the nucleus and cytoplasm. NPM is not only closely related to RNA assembly and synthesis, processing of rRNA precursors, and regulating the activity of tumor suppressor genes p53 and p14 but also participates in monitoring nucleolar activity, thus playing an important role in cell proliferation and growth [3].

The previous experiments of our group showed that NPM2 expression was significantly different between the apatinib treatment group and the control group in MPM, which provided a new idea for studying the molecular mechanism of MPM [4]. This study aimed to analyze the correlation between the expression level of NPM2 and the main clinicopathological characteristics and prognosis of MPM and to explore a new potential therapeutic target.

## Materials and methods

### Patient selection

We selected MPM patients who underwent cytoreductive surgery (CRS) plus hyperthermic intraperitoneal chemotherapy (HIPEC) in Beijing Shijitan Hospital from May 2015 to October 2021 and had complete clinical data. Specimens were collected prior to HIPEC. The selection criteria were consistent with CRS + HIPEC criteria [5].

The inclusion criteria were as follows: (1) MPM confirmed by histopathology with complete clinicopathological data and follow-up information; (2) Karnofsky performance status score (KPS) ≥ 60; (3) peripheral blood leukocytes ≥ 3.5 × 10^9^/L and platelets ≥ 80 × 10^9^/L; (4) acceptable liver function: total bilirubin, aspartate aminotransferase, alanine aminotransferase, and < 2 × upper limit of normal (ULN); (5) acceptable renal function: serum creatinine < 1.2 × ULN; and (6) heart, lung function, and other major organs can tolerate major surgery.

The exclusion criteria were as follows: (1) the lung, brain, bone, liver, and other distant metastases found on preoperative examinations, (2) obvious mesenteric contracture observed on imaging diagnosis, and (3) general physical condition and vital organs cannot withstand major operations.

The study was approved by the Medical Ethics Committee of Beijing Shijitan Hospital (2015-[28]). All patients signed an informed consent form and agreed to use postoperative specimens for medical research.

### Study parameters

The clinicopathological characteristics were sex, age, body mass index (BMI), KPS, histories of prior surgery, intravenous/intraperitoneal chemotherapy and targeted therapy, preoperative tumor markers [any one of carcinoembryonic antigen, carbohydrate antigen (CA) 19-9, CA 125, and alpha-fetoprotein], peritoneal cancer index (PCI) score, completeness of cytoreduction (CC) score, ascites, pathological type, vascular tumor emboli, lymphatic metastasis, and serious adverse events (SAEs). The immunohistochemical characteristics were Ki-67 index and NPM2 expression. The survival data were survival status and overall survival (OS).

### Immunohistochemistry (IHC) analysis

IHC was performed on 92 specimens by EnVision method. Specimens were fixed in 10% neutral formaldehyde, routinely embedded in paraffin, made 4 μm sections, deparaffinized, rehydrated in graded series of ethanol, microwaved for 10 min in citrate buffer (pH 6.0), and blocked endogenous peroxidase activity by 0.3% H_2_O_2_. Tissue sections were processed in an automated immunohistochemical staining system using standard protocols (DAKO Automated Immunohistochemical Stainer, Agilent, America). The dilutions of the IHC antibody were as follows: Ki-67 (1:100, clone UMAB107, catalog number ZM-0166, OriGene, China) and NPM2 (1:200, catalog number ab243544, Abcam, England). All antibodies were incubated for 1 h at room temperature. Positive control was used according to the instructions. PBS was used instead of the antibody as negative control.

### Histopathological quantitative analysis

**Fig. 1:**
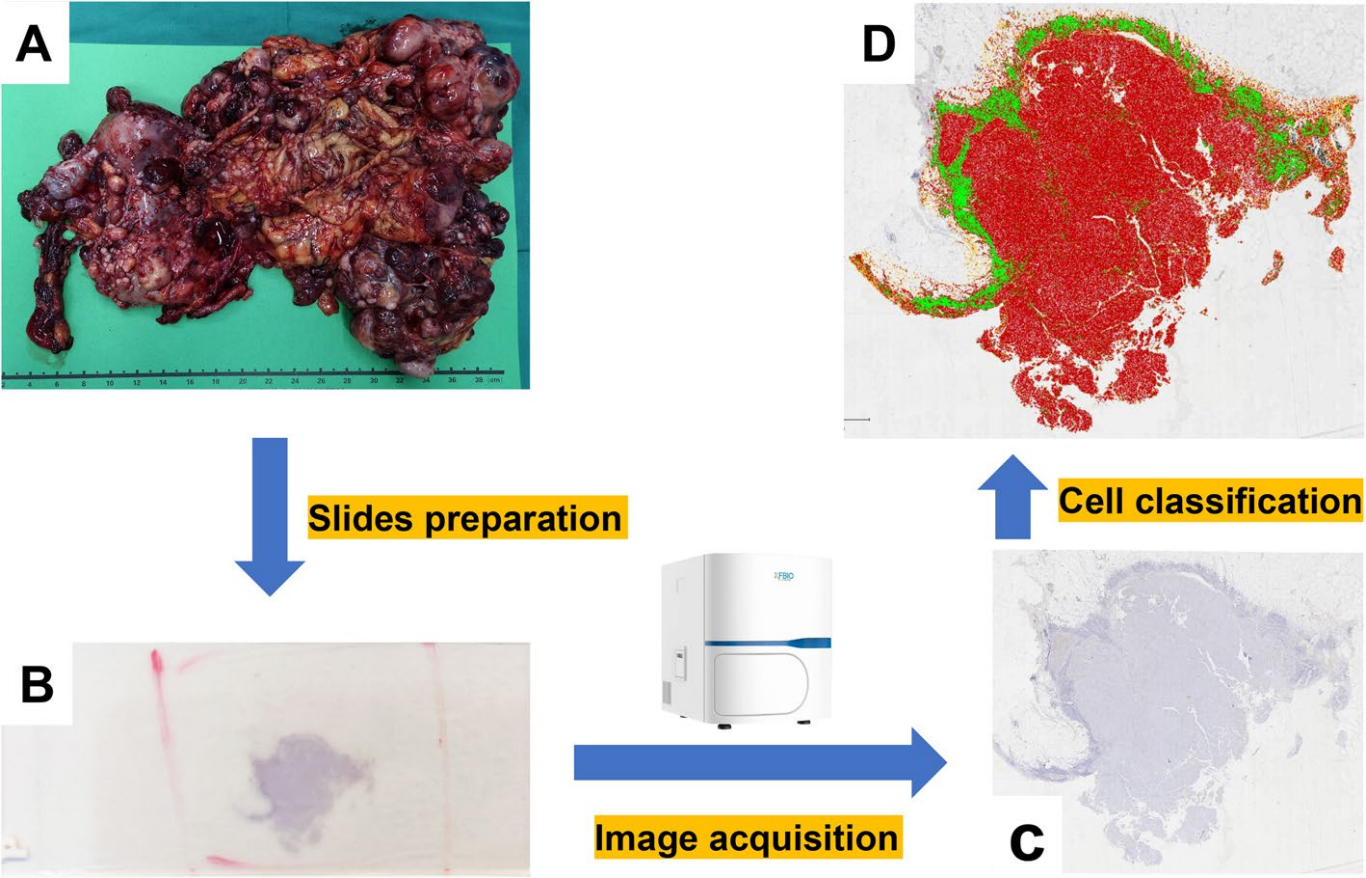
The workflow of histopathological quantitative analysis. **A** MPM surgical specimen. **B** IHC staining slide (NPM2). **C** Whole slide image. **D** Different cellular components were classified by QuPath-0.3.2 software. All four components—tumor cells (red), immune infiltrate (green), blood cells (black), and other stromal cells (yellow)—were visible on this slide (high-resolution images are presented below)

The whole process of quantitative analysis of NPM2 protein is as follows: (1) slides preparation: a senior pathologist (Du XM) selected the specimen with the most prominent tumor proliferation for IHC staining; (2) image acquisition: KF-PRO-400 scanner (Jiangfeng, China) was used for whole slide scanning; (3) format conversion: convert KFB files to SVS files using a format converter (Jiangfeng, China); and (4) machine learning: use QuPath-0.3.2 software to accurately identify tumor cells and stromal cells (Fig. 1).

### Semiquantitative scoring criteria based on QuPath-0.3.2

**Fig. 2:**
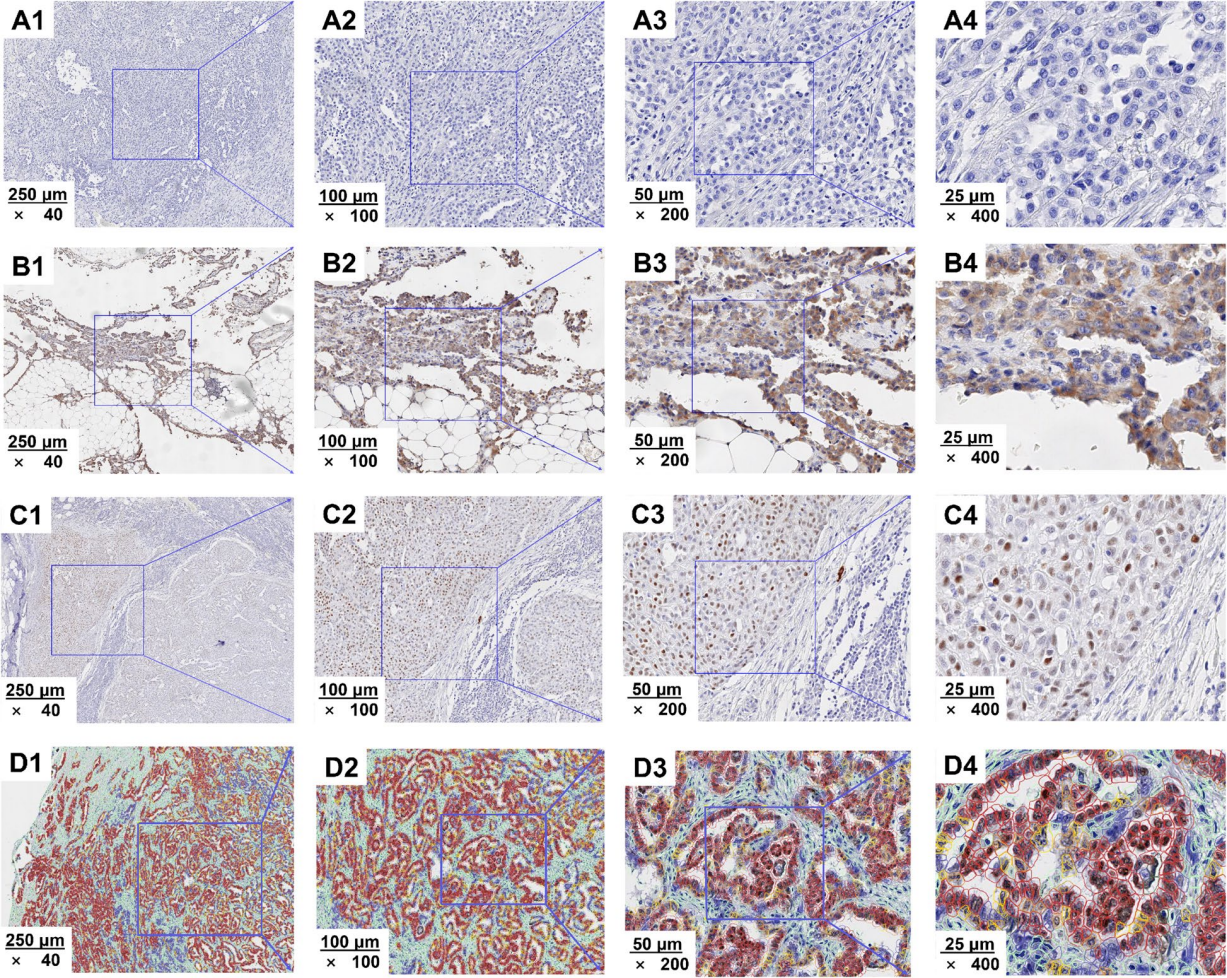
Localization and expression of NPM2 protein in MPM. **A1**–**A4** Loss of NPM2 protein expression. **B1**–**B4** NPM2 protein localized to the cytoplasm. **C1**–**C4** NPM2 protein localized to the nucleus. **D1**–**D4** Different cellular components and their staining intensity were classified by QuPath-0.3.2 software. The red, orange, yellow, and blue were all tumor cells, but the staining intensity of NPM2 protein was strongly positive, moderately positive, weakly positive, and negative, respectively. The light green was stromal cells, and the staining intensity of NPM2 protein was negative (1: ×40; 2: ×100; 3: ×200; 4: ×400)

The semiquantitative scoring criteria of NPM2 protein were as follows:

1. Positive definition: NPM2 protein was defined as positive when it appeared brown in the cytoplasm or nucleus.
2. Quantitative algorithm: “Cell: DAB OD mean” value was used to describe the staining intensity of NPM2 protein.
3. Grading standard: The “cell: DAB OD mean” values of tumor cells were collected. According to the data provided by QuPath-0.3.2 software, the staining intensity value of negative tumor cell was less than 0.1000, and the minimum and maximum staining intensity of positive cells was 0.1000 and 1.3255, respectively. Furthermore, staining intensity of positive cells was divided into 3 intervals (0–33.3%; 33.3–66.7%; 66.7–100%), according to its tri-sectional quantiles. Therefore, weak staining intensity of positive tumor cells (0–33.3%) was defined as a range of (0.1000–0.1162). Medium staining intensity of positive tumor cells (33.3–66.7%) was defined as a range of (0.1162–0.1437). Strong staining intensity of positive tumor cells (66.7–100%) was defined as a range of (0.1437–1.3255).
4. Scoring criteria: The scores of weak, medium, and strong staining intensity were 1, 2, and 3 points, respectively.
5. Comprehensive evaluation: NPM2 expression value = (number of weakly stained tumor cells/number of total tumor cells) × 1 + (number of medium stained tumor cells/number of total tumor cells) × 2 + (number of strongly stained tumor cells/number of total tumor cells) × 3; according to its median, it was divided into high or low expression of NPM2 protein (Fig. 2).

### Related definitions

NPM2 protein expression status was defined as positive or negative (loss) expression. NPM2 protein expression level was defined as negative (loss), low, or high expression. OS was defined as the time interval from the date of clinical diagnosis to the date of death or last follow-up.

### Follow-up

Follow-up records included survival status and OS. The last unified follow-up date was April 1, 2022, and the follow-up rate was 100%.

### Statistical analysis

Data analysis was performed using the Statistical Package for Social Science 25.0 software (SPSS 25.0, IBM Corporation, Armonk, NY, USA). Continuous variables were presented as mean ± SD or median (range). Classified variables were presented as numbers and percentages and analyzed using Pearson’s *χ*^2^ test or Fisher’s exact test. Multivariate correlation analyzed was conducted using logistic regression or Cox regression. OS was estimated using the Kaplan-Meier method and the log-rank test. The best boundary values of continuous variables, named with cutoff values, were determined using the Youden index of the COX curve or median. Statistical significance was set at *P* < 0.05.

## Results

### Major clinicopathological characteristics

Among the 92 MPM patients, there were 47 males (48.9%) and 45 females (51.1%), with a median age of 56 (range: 24–73). Twenty-six (28.3%) patients had *PCI* < 20, and 66 (71.7%) cases had *PCI* ≥ 20; 48 (52.2%) patients achieved CC 0/1, and 44 (47.8%) cases achieved CC 2/3. Histologically, 75 (81.5%) patients were epithelioid, and 17 (18.5%) were non-epithelioid. There were 25 (27.2%) and 67 (72.8%) patients with and without vascular tumor emboli, respectively (Table 1).

**Table 1:** Clinicopathological characteristics

Variable	Value
Gender, *n* (%)	
Male	47 (48.9)
Female	45 (51.1)
Age, *n* (%)	
< 65	75 (81.5)
≥ 65	17 (18.5)
BMI (kg/m^2^), median (range)	22.0 (15.6–32.1)
KPS, *n* (%)	
< 80	14 (15.2)
≥ 80	78 (84.8)
History of prior surgery, *n* (%)	
No	38 (41.3)
Yes	54 (58.7)
History of IV/IP chemotherapy, *n* (%)	
No	45 (48.9)
Yes	47 (51.1)
History of targeted therapy, *n* (%)	
No	65 (70.7)
Yes	27 (29.3)
Increased preoperative TMs^a^, *n* (%)	
No	28 (30.4)
Yes	64 (69.6)
PCI score, *n* (%)	
< 20	26 (28.3)
≥ 20	66 (71.7)
CC score, *n* (%)	
0/1	48 (52.2)
2/3	44 (47.8)
Ascites (mL), *n* (%)	
0	19 (20.7)
0–1000	16 (17.4)
> 1000	57 (62.0)
Pathological type, *n* (%)	
Epithelioid	75 (81.5)
Non-epithelioid	17 (18.5)
Vascular tumor emboli, *n* (%)	
No	67 (72.8)
Yes	25 (27.2)
Lymphatic metastasis, *n* (%)	
No	82 (89.1)
Yes	10 (10.9)
Ki-67 index, *n* (%)	
≤ 9%	15 (16.3)
> 9%	77 (83.7)
SAEs, *n* (%)	
No	63 (68.5)
Yes	29 (31.5)

*BMI* body mass index, *KPS* Karnofsky performance status score, *PSS* prior surgical scores, *IV/IP* intravenous/intraperitoneal, *TMs* tumor markers. ^a^Any one of carcinoembryonic antigen, carbohydrate antigen (CA)19-9, CA 125, and alpha-fetoprotein was increased. *PCI* peritoneal cancer index, *CC* completeness of cytoreduction, *SAEs* serious adverse events

### Localization and expression of NPM2 protein

#### Localization of NPM2 protein

The localization of NPM2 protein was divided into three types: (1) the expression NPM2 protein was negative (70/92, 76.1%); (2) NPM2 protein localized to the nucleus (4/92, 4.3%); and (3) NPM2 protein localized to the cytoplasm (18/92, 19.6%). Due to the small sample sizes in this study, the differences of localization in the nucleus (4/22, 18.2%) and cytoplasm (18/22, 81.2%) could not be analyzed (Fig. 2).

#### Expression of NPM2 protein

Among the 92 MPM patients, 22 (23.9%) were positive for NPM2 protein expression, and 70 (76.1%) were negative. Among the 22 cases with positive NPM2 protein expression, the median expression value of NPM2 protein was 0.41314 (range: 0.02397–2.72636). Eleven cases (50%) had high NPM2 protein expression level (≥ 0.41314), and 11 cases (50%) had low expression level (< 0.41314).

### Correlation analysis between NPM2 protein and clinicopathological features of MPM

#### The relationship between NPM2 protein expression and clinicopathological features of MPM

Univariate analysis showed that NPM2 protein expression was negatively correlated with the following 4 clinicopathological factors: PCI score (*χ*^2^ = 4.216, *P* = 0.040), CC score (*χ*^2^ = 4.895, *P* = 0.027), and vascular tumor emboli (*χ*^2^ = 7.481, *P* = 0.006), SAEs (*χ*^2^ = 4.285, *P* = 0.038). Factors in the univariate survival analysis (*P* < 0.05) were incorporated into the Cox regression model for multivariate analysis. The results showed that NPM2 protein level expression (negative vs. positive) was independently negatively correlated with the following two clinicopathological factors: CC score [odds ratio (OR) = 0.332, 95% *CI*: 0.112–0.984, *P* = 0.047] and vascular tumor emboli (*OR* = 0.095, 95% *CI* = 0.012–0.764, *P* = 0.027) (Table 2).

**Table 2:** Correlation between NPM2 expression status (negative vs. positive) and clinicopathological characteristics of MPM

Variable	***n*** (%)	NPM2 status (***n***, %)		***P***
		Negative	Positive	
Gender				0.274
Male	47 (48.9)	38 (41.3)	9 (9.8)	
Female	45 (51.1)	32 (34.8)	13 (14.1)	
Age				0.556
< 65	75 (81.5)	58 (63.0)	17 (18.5)	
≥ 65	17 (18.5)	12 (13.0)	5 (5.4)	
History of targeted therapy				0.172
No	65 (70.7)	52 (56.5)	13 (14.1)	
Yes	27 (29.3)	18 (19.6)	9 (9.8)	
Increased preoperative TMs^a^				0.079
No	28 (30.4)	18 (19.6)	10 (10.9)	
Yes	64 (69.6)	52 (56.5)	12 (13.0)	
Ascites (mL)				
0	19 (20.7)	12	7	0.175
0–1000	16 (17.4)	11	5	
> 1000	57 (62.0)	47	10	
PCI score				**0.040**
< 20	26 (28.3)	16 (17.4)	10 (10.9)	
≥ 20	66 (71.7)	54 (58.7)	12 (13.0)	
CC score				**0.027**
0/1	48 (52.2)	32 (34.8)	16 (17.4)	
2/3	44 (47.8)	38 (41.3)	6 (6.5)	
Ki-67 index				0.054
≤ 9%	15 (16.3)	8 (8.7)	7 (7.6)	
> 9%	77 (83.7)	62 (67.4)	15 (16.3)	
Vascular tumor emboli				**0.006**
No	67 (72.8)	46 (50.0)	21 (22.8)	
Yes	25 (27.2)	24 (26.1)	1 (1.1)	
SAEs				**0.038**
No	63 (68.5)	44 (47.8)	19 (20.7)	
Yes	29 (31.5)	26 (28.3)	3 (3.3)	

*TMs* tumor markers. ^a^Any one of carcinoembryonic antigen, carbohydrate antigen (CA) 19-9, CA 125, and alpha-fetoprotein was increased. *PCI* peritoneal cancer index, *CC* completeness of cytoreduction, *SAEs* serious adverse events

#### The relationship between NPM2 protein expression level and clinicopathological features of MPM

Univariate analysis showed that NPM2 protein expression level (negative vs. low expression vs. high expression) was negatively correlated with the following three clinicopathological factors: CC score (*χ*^2^ = 7.810, *P* = 0.020), vascular tumor emboli (*χ*^2^ = 10.927, *P* = 0.004), and SAEs (*χ*^2^ = 9.420, *P* = 0.009). Factors in the univariate survival analysis (*P* < 0.05) were incorporated into the Cox regression model for multivariate analysis. The results showed that NPM2 protein expression level (negative vs. low expression vs. high expression) was independently negatively correlated with the following two clinicopathological factors: CC score (*OR* = 0.317, 95% *CI*: 0.317–0.959, *P* = 0.042) and vascular tumor emboli (*OR* = 0.092, 95% *CI* = 0.011–0.770, *P* = 0.028) (Table 3).

**Table 3:** Correlation between NPM2 expression level (negative vs. low expression vs. high expression) and clinicopathological characteristics of MPM

Variable	***n*** (%)	NPM2 expression level (***n***, %)			***P***
		Negative	Low	High	
Gender					0.501
Male	47 (48.9)	38 (41.3)	5 (5.4)	4 (5.6)	
Female	45 (51.1)	32 (34.8)	6 (6.5)	7 (7.6)	
Age					0.723
< 65	75 (81.5)	58 (63.0)	8 (8.7)	9 (9.8)	
≥ 65	17 (18.5)	12 (13.0)	3 (3.3)	2 (2.2)	
History of targeted therapy					0.173
No	65 (70.7)	52 (56.5)	5 (5.4)	8 (8.7)	
Yes	27 (29.3)	18 (19.6)	6 (6.5)	3 (3.3)	
Increased preoperative TMs^a^					0.139
No	28 (30.4)	18 (19.6)	4 (4.3)	6 (6.5)	
Yes	64 (69.6)	52 (56.5)	7 (7.6)	5 (5.4)	
Ascites (mL)					
0	19 (20.7)	12 (13.0)	2 (2.2)	5 (5.4)	0.256
0–1000	16 (17.4)	11 (12.0)	3 (3.3)	2 (2.2)	
> 1000	57 (62.0)	47 (51.1)	6 (6.5)	4 (4.3)	
PCI score					0.094
< 20	26 (28.3)	16 (17.4)	4 (4.3)	6 (6.5)	
≥ 20	66 (71.7)	54 (58.7)	7 (7.6)	5 (5.4)	
CC score					**0.020**
0/1	48 (52.2)	32 (34.8)	6 (6.5)	10 (10.9)	
2/3	44 (47.8)	38 (41.3)	5 (5.4)	1 (1.1)	
Ki-67 index					0.093
≤ 9%	15 (16.3)	8 (8.7)	4 (4.3)	3 (20.0)	
> 9%	77 (83.7)	62 (67.4)	7 (7.6)	8 (8.7)	
Vascular tumor emboli					**0.004**
No	67 (72.8)	46 (50.0)	10 (10.9)	11 (12.0)	
Yes	25 (27.2)	24 (26.1)	1 (1.1)	0 (0.0)	
SAEs					**0.009**
No	63 (68.5)	44 (47.8)	11 (12.0)	8 (8.7)	
Yes	29 (31.5)	26 (28.3)	0 (0.0)	3 (3.3)	

*TMs* tumor markers. ^a^Any one of carcinoembryonic antigen, carbohydrate antigen (CA) 19-9, CA 125, and alpha-fetoprotein was increased. *PCI* peritoneal cancer index, *CC* completeness of cytoreduction, *SAEs* serious adverse events

### Survival analysis

#### Overall survival curve analysis

**Fig. 3:**
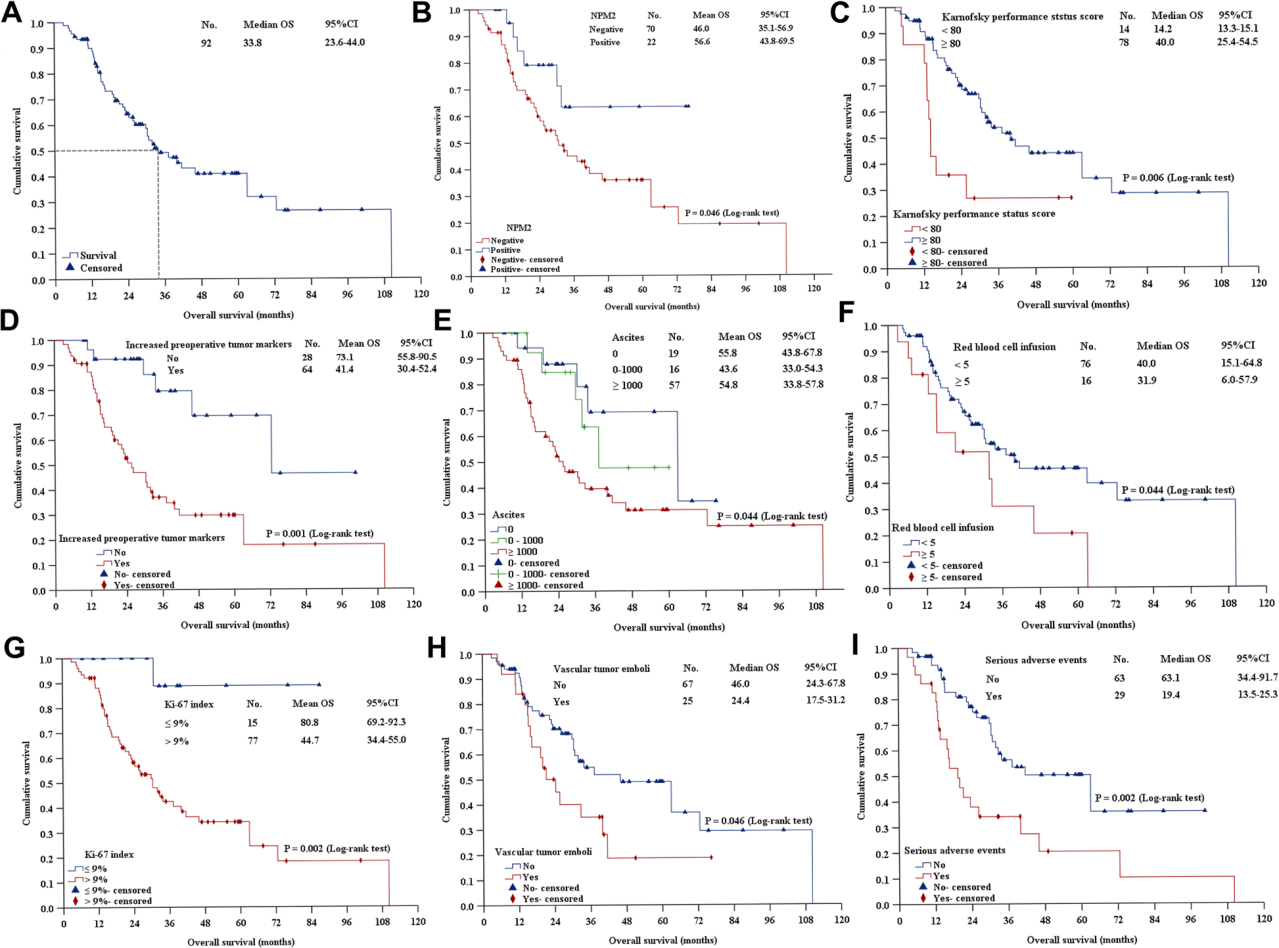
Survival analysis. **A** Overall survival. **B** NPM2 protein expression. **C** Karnofsky performance status score. **D** Increased preoperative tumor markers (any one of carcinoembryonic antigen, carbohydrate antigen (CA) 19-9, CA 125, and alpha-fetoprotein was increased). **E** Ascites. **F** Red blood cell infusion. **G** Ki-67. **H** Vascular tumor emboli. **I** Serious adverse events

The median follow-up time was 46.9 months (95% *CI*: 35.8–57.9 months), and the median overall survival (OS) was 33.8 months (95% *CI*: 23.6–44.0 months). Forty-eight patients (52.2%) were died, and 44 (47.8%) were survived. The 1-, 2-, 3-, and 5-year survival rates were 90.0%, 64.4%, 49.2%, and 41.0%, respectively (Fig. 3A).

#### Univariate analysis

Univariate survival analysis showed that the prognosis of MPM was related to the following eight clinicopathological factors: NPM2 protein expression (*P* = 0.046), KPS (*P* = 0.006), increased preoperative tumor markers (*P* = 0.001), ascites (*P* = 0.044), red blood cell infusion (*P* = 0.044), Ki-67 (*P* = 0.002), vascular tumor emboli (*P* = 0.046), and SAEs (*P* = 0.002). However, NPM2 protein expression level was not associated with MPM prognosis (*P* > 0.05) (Fig. 3 B–I).

#### Multivariate analysis

Factors in the univariate survival analysis (*P* < 0.05) were incorporated into the Cox regression model for multivariate analysis, delineating the following five independent prognostic factors: KPS [hazard rate (HR) = 0.321, 95% *CI*: 0.147–0.700, *P* = 0.004], preoperative tumor markers (*HR* = 3.604, 95% *CI*: 1.512–8.591, *P* = 0.004), Ki-67 (*HR* = 10.603, 95% *CI*: 1.117–77.700, *P* = 0.020), SAEs (*HR* = 2.122, 95% *CI*: 1.003–4.307, *P* = 0.013), and red blood cell infusion (*HR* = 2.079, 95% *CI*: 1.003–4.307, *P* = 0.049). However, NPM2 protein expression was not an independent prognostic factor in MPM.

## Discussion

In this study, QuPath-0.3.2 software was used to quantitatively analyze the expression level of NPM2 protein in whole slide images [6]. The results showed the following: (1) NPM2 expression status was negatively correlated with the following 4 clinicopathological factors: PCI score, CC score, vascular tumor emboli, and SAEs. NPM2 expression level was negatively correlated with the following three clinicopathological factors: CC score, vascular tumor emboli, and SAEs. NPM2 expression level was independently negatively correlated with the vascular tumor emboli. (2) NPM2 protein is mostly localized in the cytoplasm. (3) Loss of NPM2 expression was associated with poor prognosis. However, NPM2 expression level was not associated with the prognosis of MPM.

NPM is located on chromosome 5q35 and includes subtypes NPM1, NPM2, and NPM3 [7]. Wild-type NPM can shuttle between the nucleus and cytoplasm through nuclear pores. The shuttle process is regulated by nuclear export signal (NES), nuclear localization signal (NLS), and nucleolar localization signal (NoLS) [8–10]. Research on NPM mainly focus on NPM1, and there is a lack of related reports on NPM2. In adult acute myeloid leukemia (AML), one-third of patients develop mutations in the NPM1 gene, resulting in abnormal cytoplasmic localization [11]. Due to its unique features, NPM1-mutated AML is recognized as a distinct entity in the 2017 WHO classification of hematopoietic neoplasms [10, 12]. In 2017, the consensus released by the European LeukemiaNet showed that NPM1 mutations convey a relatively favorable prognosis when FLT3-ITD is absent or shows a low allelic ratio (< 0.5, FLT3-ITD^low^) [13]. Our results showed that NPM2 protein was mostly localized in the cytoplasm (18/22, 81.8%) with a better prognosis, suggesting the possibility of mutations for NPM2 gene. Since the NPM sequence mutation has not been observed in solid tumors, further experiments are needed to investigate whether the NPM2 gene is mutated in MPM [10].

During tumor initiation and promotion, whether NPM is a proto-oncogene or a tumor suppressor gene has been controversial. In a variety of solid tumors (gastric cancer, colon cancer, and thyroid cancer), NPM1 overexpression can directly participate in tumorigenesis, and the expression level of NPM1 is positively correlated with tumor development stage [14–16]. Currently, no amplification of the NPM1 gene has been detected in human tumors, but the gene is deleted in myelodysplastic syndromes [17]. Meanwhile, NPM is also involved in embryonic development. Complete deletion of NPM is lethal in mouse mid-gestation, and embryos display genomic instability, extensive apoptosis, and p53 activation [18]. More importantly, NPM haploinsufficiency in NPM+/− mouse embryonic fibroblasts that mimic cancer cells harboring chromosomal rearrangements/deletions at the NPM1 locus shows an immortal phenotype with high proliferation [19]. NPM+/− mice are more prone to malignancies than wild-type mice, especially of hematologic and lymphoid origin, suggesting that NPM is a haploinsufficient tumor suppressor [20]. Karhemo et al. [21] found that low NPM1 expression was associated with poor prognosis in breast cancer (*n* = 1160). Fujiwara et al. [22] found that the expression of NPM2 protein was significantly different in normal melanocytes and malignant melanoma cells, which were 74.6% (50/67) and 15.6% (5/32), respectively. The results showed that there was a significant loss of NPM2 protein expression in malignant melanoma cells. Therefore, NPM2 protein expression could distinguish malignant melanoma from normal melanocytes. Our study suggested that 76.1% of patients lacked NPM2 protein expression with poor prognosis, suggesting that NPM2 may be a tumor suppressor gene in MPM. However, a 5-year survival of pleura mesothelioma patients with low-NPM2 expression was higher than that of the high-NPM2-expression pleura mesothelioma patients, a result different from our current study [4]. There are three possible reasons. First, the types of mesotheliomas were different in the two experiments, one pleural mesothelioma and one peritoneal mesothelioma. Second, the races of patients were different, one predominantly white and one predominantly yellow people. Finally, the methods were different, one RNA sequencing and one IHC. Further research was needed.

Previous study had shown that PCI score, CC score, vascular tumor embolic, and SAEs were related factors affecting the prognosis of MPM [5]. Our study showed that the expression level of NPM2 protein was negatively correlated with PCI score, CC score, vascular tumor embolic, and SAEs. It is particularly noteworthy that the level of NPM2 expression was independently negatively correlated with the vascular tumor emboli, indicating that NPM2 could help evaluate the biological behavior of MPM.

The limitation of this study is that the sample sizes were small. It is necessary to conduct a multicenter study with larger sample sizes to provide higher-level evidence for clarifying the clinical application value of NPM2 in MPM.

## Conclusion

This study found that NPM2 protein is mostly localized in the cytoplasm, suggesting the possibility of mutations for NPM2 gene. A total of 76.1% of patients lacked NPM2 protein expression with poor prognosis, suggesting that NPM2 may be a tumor suppressor gene. NPM2 expression level was independently negatively correlated with the vascular tumor emboli, indicating that NPM2 could help evaluate the biological behavior of MPM. Therefore, loss of NPM2 expression is a potential immunohistochemical marker for MPM.

## Data Availability

The datasets used and/or analyzed during the current study are available from the corresponding author on reasonable request

## Abbreviations

NPM2: Nucleoplasmin 2
MPM: Malignant peritoneal mesothelioma
WHO: World Health Organization
CRS: Cytoreductive surgery
HIPEC: Hyperthermic intraperitoneal chemotherapy
KPS: Karnofsky performance status score
BMI: Body mass index
CA: Carbohydrate antigen
PCI: Peritoneal cancer index
CC: Completeness of cytoreduction
SAEs: Serious adverse events
OS: Overall survival
NES: Nuclear export signal
NLS: Nuclear localization signal
NoLS: Nucleolar localization signal
AML: Acute myeloid leukemia
